# Omega-3 supplementation during pregnancy in relation to reported information sources: an exploratory cross-sectional study

**DOI:** 10.1186/s12884-026-10082-8

**Published:** 2026-10-12

**Authors:** Anna Elisabeth Hentrich, Doerthe Brueggmann, Samira Catharina Hoock, Lukas Jennewein, Nina-Naomi Kreis, Wiebke Schaarschmidt, Anne Kristina Kaempf, Frank Louwen, Eileen Deuster

**Affiliations:** Department of Obstetrics and Perinatal Medicine, University Hospital, Goethe University Frankfurt, 60590 Frankfurt, Germany

**Keywords:** Omega-3 fatty acids, Pregnancy, Supplementation, Parity, Health communication, Maternal nutrition

## Abstract

**Background:**

Omega-3 fatty acid supplementation during pregnancy is recommended in certain settings and may be relevant for maternal and fetal health, yet uptake varies widely. While educational attainment is commonly considered a determinant of health behavior, the relationship between reported information sources and supplementation practices during pregnancy remains insufficiently understood.

**Methods:**

A cross-sectional online survey was conducted among pregnant women attending a university hospital in Germany between June 2024 and January 2025. Data were collected on current omega-3 supplementation, preconception omega-3 use, educational attainment, parity, and reported sources of information about dietary supplements. Source-specific associations with current and preconception omega-3 use were examined exploratorily using 2 × 2 contingency tables. Conditional maximum-likelihood odds ratios (ORs) with central exact 95% confidence intervals (CIs) and corresponding two-sided central exact P values were calculated.

**Results:**

A total of 127 completed questionnaires were included. Overall, 75.6% of participants reported current omega-3 supplementation, while 34.6% reported preconception omega-3 use. The internet was the most frequently reported source of information about dietary supplements (73.2%), followed by gynecologists (56.7%) and books (37.0%). Participants reporting midwives as an information source had higher estimated odds of current omega-3 supplementation (OR 6.84, 95% CI 1.56–62.84). Participants reporting digital/media sources had higher estimated odds of preconception omega-3 use (OR 6.59, 95% CI 1.47–61.13). Both estimates were imprecise, with wide confidence intervals. No clear association between educational attainment and omega-3 supplementation was observed in this highly educated sample.

**Conclusions:**

In this selective sample, exploratory analyses identified patterns of co-occurrence between reported sources of information about dietary supplements and omega-3 supplementation behavior. The findings do not establish that specific information sources provided omega-3-specific advice, influenced supplementation behavior, or preceded omega-3 use. Larger prospective studies are needed to examine these relationships and the content and timing of information exposure.

**Supplementary Information:**

The online version contains supplementary material available at https://doi.org/10.1186/s12884-026-10082-8.

## Introduction

Maternal nutrition has a well-documented impact on fetal development. Long-chain omega-3 fatty acids, particularly docosahexaenoic acid (DHA), are important structural components of the developing fetal brain and retina [1, 2]. Observational studies and randomized trials have suggested potential benefits of adequate omega-3 intake during pregnancy for neurodevelopmental and visual outcomes in offspring; however, findings across studies remain heterogeneous [3]. Evidence for omega-3 supplementation during pregnancy also varies according to the outcome assessed, baseline maternal omega-3 status, dietary intake, supplementation dose, and population characteristics. The strongest evidence has been reported for a reduction in the risk of preterm birth, particularly among women with lower baseline omega-3 intake or status. In contrast, evidence regarding cognitive development, visual function, postpartum depression, and preeclampsia remains less consistent, and reported effects differ across studies and populations [4–7].

Despite the growing body of evidence supporting omega-3 supplementation during pregnancy, implementation of recommendations remains inconsistent. The German Nutrition Society (DGE) recommends regular consumption of fatty fish and, when dietary intake is insufficient, supplementation with at least 200 mg DHA per day [1]. Similar recommendations have been issued by international expert groups and organizations, including the European Food Safety Authority [8], the International Society for the Study of Fatty Acids and Lipids (ISSFAL) [2], and the International Federation of Gynecology and Obstetrics (FIGO) [9], although recommended intake levels and implementation strategies vary slightly between organizations. In contrast, the World Health Organization (WHO) emphasizes adequate maternal nutrition and a balanced diet during pregnancy rather than recommending routine omega-3 supplementation for all pregnant women. Nevertheless, actual omega-3 intake and supplementation practices vary considerably among pregnant women and appear to be influenced by sociodemographic characteristics, health beliefs, access to information, and individual health behaviors [10–12].

Previous studies have shown that awareness, knowledge, and use of omega-3 supplements during pregnancy vary considerably across populations. Research from Australia and other countries has demonstrated that many pregnant women are aware of recommendations regarding omega-3 intake but often have limited knowledge regarding specific dietary sources, recommended intake levels, and potential health effects [13, 14]. Other studies have reported that supplement use during pregnancy is associated with sociodemographic factors including maternal age, educational attainment, parity, and socioeconomic status [11, 15]. Information sources have also been associated with maternal nutrition behavior, with healthcare professionals, family networks, and digital resources representing commonly reported sources of nutrition-related information during pregnancy [10, 16]. Recent German studies further indicate substantial use of dietary supplements during pregnancy, while highlighting persistent variation in nutritional knowledge and in the implementation of recommendations [17, 18].

Furthermore, many women do not begin supplementation until after pregnancy has been established, despite the potential benefits of adequate nutritional preparation during the preconception period [14, 19]. Understanding factors that influence both supplementation uptake during pregnancy and preconception omega-3 use may therefore be important for improving adherence to nutritional recommendations.

Seeking health information has become increasingly complex in recent years. Pregnant women obtain nutrition-related information from healthcare professionals, family and social networks, printed materials, and a rapidly expanding range of digital information sources. Previous studies have demonstrated that internet-based resources are among the most frequently used sources of pregnancy-related health information [20–23]. More recent evidence from Germany similarly indicates that online resources are among the most commonly used channels for obtaining nutrition-related information during pregnancy [18]. At the same time, healthcare professionals remain important providers of nutrition-related counselling and pregnancy-specific recommendations [15]. However, while the use of different information sources has been described previously, their relationship with actual supplementation behavior remains incompletely understood.

In addition to formal educational attainment, health literacy and information-seeking behavior may represent important determinants of health-related decision-making during pregnancy [24–27]. Previous studies have shown that educational attainment alone does not necessarily predict the ability to identify, interpret, and apply evidence-based health information. Consequently, both the source of information and the capacity to evaluate information may influence adherence to nutritional recommendations.

The present study aimed to describe omega-3 supplementation during pregnancy and to explore whether reported information sources, educational attainment, and parity were associated with supplementation behavior in a selective university-hospital-based sample. In addition, we explored whether reported information sources were associated with preconception omega-3 use. Given the cross-sectional design, small sample size, and exploratory nature of the analyses, no confirmatory hypotheses were tested.

## Methods

### Study design and participants

A cross-sectional online survey was conducted between June 2024 and January 2025 at the University Hospital Frankfurt am Main. Pregnant women were recruited either during bi-monthly hospital-based parental information evenings or outpatient antenatal visits. Participation was voluntary and anonymous. The survey was accessed via a QR code, and ethical approval was granted with a waiver for anonymous data collection (Ethics Committee of the Johann Wolfgang Goethe-University Hospital Frankfurt number 2024 − 1732).

### Survey instrument

A standardized questionnaire was developed and pre-tested by pregnant women, physicians, and psychologists to ensure clarity and content validity. The survey collected data on sociodemographic characteristics (age, educational level, parity), dietary supplement use, omega-3 supplementation behavior, preconception omega-3 use, knowledge of supplement ingredients, and sources of information. Omega-3 supplementation was assessed as current self-reported use during pregnancy (yes/no). The questionnaire did not collect information on supplement dosage, DHA/EPA content, product formulation, frequency of intake, duration of use, or adherence. Consequently, the outcome reflects self-reported omega-3 supplement use and should not be interpreted as guideline-concordant supplementation. Current omega-3 supplementation was derived from questionnaire item 13, “Are you taking Omega-3?”, with the response options “Yes” and “No”. Participants selecting “Yes” were classified as reporting current omega-3 supplementation, whereas participants selecting “No” were classified as not reporting current omega-3 supplementation.

Preconception omega-3 use was derived from questionnaire item 14, “Did you take omega-3 supplements before pregnancy?”. Response options were “Yes, for more than 3 months”, “Yes, for more than 4 weeks”, “Yes, starting at the beginning of pregnancy”, “Yes, on medical advice since early pregnancy”, and “No, I do not take omega-3 supplements”. Participants selecting “Yes, for more than 3 months” or “Yes, for more than 4 weeks” were classified as reporting preconception omega-3 use. All remaining response categories were classified as not reporting preconception omega-3 use. Thus, the endpoint was defined as preconception omega-3 use versus all other participants and not as timing of initiation among omega-3 users.

Educational level was categorized as low, medium, or high according to the International Standard Classification of Education (ISCED 2011). Low corresponded to ISCED levels 0–2 (lower secondary school certificate), medium to ISCED levels 3–4 (intermediate secondary school certificate, vocational qualification, or university entrance qualification), and high to ISCED levels 5–8 (Bachelor’s degree, Master’s/Magister degree, or state examination) [28]. Parity was recorded as the number of previous live births and analyzed both as a binary variable (nulliparous vs. multiparous) and as an ordered categorical variable (0, 1, and ≥ 2 previous live births) for exploratory analyses. Preconception omega-3 supplementation was defined as self-reported use before pregnancy (i.e., more than 4 weeks or more than 3 months before pregnancy). Information sources were assessed as a multiple-response item, allowing participants to select more than one source. The item referred to sources of information about dietary supplements in general and did not assess whether a particular source had provided omega-3-specific information or recommended omega-3 supplementation. Individual sources included midwives, gynecologists, friends, family members, internet sources, books, magazines, social media, information evenings, pharmacies, and other sources. Sources were additionally grouped into three predefined composite categories: professional sources (midwife, gynecologist, or pharmacy), informal sources (friends or family), and digital/media sources (internet or social media). A participant was classified as having used a given composite source if she reported at least one of its constituent individual sources. These composite categories were analyzed both descriptively (Table 4) and as exploratory inferential variables (Table 5); as with the individual-source analyses, associations involving composite categories should be interpreted as exploratory. The survey also assessed participants’ perceived sufficiency of dietary omega-3 intake and their knowledge regarding the primary role of omega-3 fatty acids in fetal development.

This analysis is part of a larger cross-sectional study on maternal micronutrient intake, focusing here specifically on omega-3 supplementation.

### Data analysis

Descriptive statistics were used to summarize participant characteristics, omega-3 supplementation behavior, knowledge, and reported information sources. Categorical variables are presented as frequencies and percentages, whereas continuous variables are presented as means with standard deviations (SDs).

Associations between omega-3 supplementation and educational attainment, parity, and reported information sources were assessed using chi-square tests or exact tests, as appropriate. Because information sources were assessed using a multiple-response item, each individual information source and each of the three predefined composite source categories was analyzed separately as a binary variable (reported vs. not reported).

For the source-specific analyses, associations with current omega-3 supplementation and preconception omega-3 use were evaluated using 2 × 2 contingency tables. Given the small sample size and sparse cells, odds ratios (ORs) were estimated using conditional maximum likelihood and are presented with central exact 95% confidence intervals (CIs). Corresponding two-sided central exact P values were calculated within the same conditional framework. ORs were consistently parameterized to compare participants reporting the respective information source with those not reporting that source, with values > 1 indicating higher odds of the respective omega-3 outcome among participants reporting the source.

Because multiple, partly overlapping information-source variables were examined, all source-specific analyses were considered exploratory. No adjustment for multiple comparisons was applied. Interpretation therefore focused primarily on the magnitude and precision of the effect estimates, while P values were reported descriptively without applying a dichotomous threshold for statistical significance.

Effect sizes for other categorical associations were reported using Phi (φ) or Cramer’s V, depending on table size. For the binary parity analysis, Fisher’s exact test was used. For the three-category parity analysis, the Fisher–Freeman–Halton exact test was used because of sparse cell counts. Because parity represents an ordered variable, an exploratory linear-by-linear association test was additionally performed across parity categories, for which the corresponding two-sided exact P value is reported. Owing to the limited sample size and sparse subgroup structure, multivariable regression modelling was not included in the final analysis.

Descriptive analyses, contingency tables, chi-square tests, exact tests, and effect-size analyses were performed using IBM SPSS Statistics version 29.0 (IBM Corp., Armonk, NY, USA). Conditional maximum-likelihood ORs, central exact 95% CIs, and corresponding two-sided central exact P values for the source-specific 2 × 2 analyses were calculated from the SPSS-derived contingency-table counts using Python with SciPy version 1.17.0.

## Results

### Sample characteristics

Of 296 QR code accesses, 143 surveys were started. After exclusion of incomplete questionnaires, 127 completed surveys were included in the final analysis. Participants were predominantly between 25 and 35 years of age (69.3%), with 26.8% aged 36–45 (Table 1). Most participants were nulliparous (79.5%), whereas 17.3% had one previous live birth and 3.1% had two or more previous live births. Educational attainment was high overall, with 93.7% of participants classified as having a high level of education.

**Table 1:** Descriptive Analysis. Percentages are based on available responses.

Variable	Category	*n* (%)
Age	< 25 years	1 (0.8%)
	25–35 years	88 (69.3%)
	36–45 years	34 (26.8%)
	> 45 years	2 (1.6%)
Parity	0 children	101 (79.5%)
	1 child	22 (17.3%)
	≥ 2 children	4 (3.1%)
Educational level	High	119 (93.7%)
	Medium	6 (4.7%)
	Low	2 (1.6%)

Missing values: age (*n* = 2)

### Supplementation behavior

Overall, 75.6% (*n* = 96) of participants reported taking omega-3 supplements during pregnancy. Overall, 44 participants (34.6%) were classified as reporting preconception omega-3 use (Table 2).

Regarding dietary sufficiency of omega-3, 46.5% believed diet alone was adequate, 34.6% disagreed, and 18.1% were unsure. When asked about the role of omega-3 in fetal development, 62.2% identified brain development as the primary role. Other responses included bone development (30.7%) and nervous system development (7.1%).

**Table 2:** Omega-3 supplementation behavior and knowledge. Percentages are based on available responses.

Variable	Category	*n* (%)
Current omega-3 supplementation	Yes	96 (75.6)
	No	31 (24.4)
Preconception omega-3 use	Yes	44 (34.6)
	No	83 (65.4)
Dietary intake alone sufficient	Yes	59 (46.5)
	No	44 (34.6)
	Unsure	23 (18.1)
Knowledge of omega-3 function	Brain development	79 (62.2)
	Bone development	39 (30.7)
	Nervous system development	9 (7.1)

Missing values: perceived dietary sufficiency (*n* = 1)

### Educational attainment

Current omega-3 supplementation was reported by 76.5% of participants with high educational attainment and 62.5% of those with medium/low educational attainment. The estimate was imprecise given the small number of participants in the medium/low education group (two-sided Fisher’s exact *P* = 0.403; φ = 0.08) (Table 3).

**Table 3:** Participant characteristics associated with current omega-3 supplementation.

Variable	Category	Supplementation Yes *n* (%)	Supplementation No *n* (%)	*p*-value	Effect size
Educational level	High	91 (76.5)	28 (23.5)	0.403¹	φ = 0.08
	Medium/Low	5 (62.5)	3 (37.5)		
Parity (binary)	Nulliparous	81 (80.2)	20 (19.8)	0.023²	φ = 0.21
	Multiparous	15 (57.7)	11 (42.3)		
Parity (3 categories)	0 children	81 (80.2)	20 (19.8)	0.050³	V = 0.21
	1 child	13 (59.1)	9 (40.9)		
	≥ 2 children	2 (50.0)	2 (50.0)		
Linear trend	Increasing parity	–	–	0.022⁴	–

¹ Educational attainment was grouped as high versus medium/low according to the predefined classification; a two-sided Fisher’s exact P value is reported

² for binary parity, a two-sided Fisher’s exact P value is reported

³ for the three-category parity analysis, the two-sided Fisher–Freeman–Halton exact P value is reported; Cramer’s V is presented as the measure of association

⁴ Two-sided exact P value for the linear-by-linear association

Values are presented as n (%), with percentages calculated within each category. Analyses were exploratory, and P values were interpreted descriptively without applying a dichotomous threshold for statistical significance

### Parity

When parity was analyzed as a binary variable, current omega-3 supplementation was reported by 80.2% of nulliparous participants and 57.7% of multiparous participants (two-sided Fisher’s exact *P* = 0.023; φ = 0.21). When examined across three ordered parity categories (0, 1, and ≥ 2 previous live births), current omega-3 supplementation was reported by 80.2%, 59.1%, and 50.0% of participants, respectively. The Fisher–Freeman–Halton exact P value was 0.050 (Cramer’s V = 0.21). An exploratory linear-by-linear analysis indicated a decreasing pattern of current omega-3 supplementation across increasing parity categories (two-sided exact *P* = 0.022). Given the small number of participants with ≥ 2 previous live births (*n* = 4), these findings should be interpreted cautiously (Table 3).

### Information sources

Participants reported multiple information sources. The most frequently reported source was the internet (73.2%), followed by gynecologists (56.7%), books (37.0%), and midwives (26.0%). Social media (18.1%), family members (12.6%), magazines (8.7%), pharmacies (8.7%), and other sources (6.3%) were reported less frequently. When grouped descriptively, digital/media sources were reported by 82.7% of participants, professional sources by 66.9%, and informal sources by 26.0% (Table 4).

**Table 4:** Reported sources of information about dietary supplements during pregnancy (multiple responses possible).

Information source	*n* (%)
Internet	*93 (73.2)*
Gynecologist	*72 (56.7)*
Books	*47 (37.0)*
Midwife	*33 (26.0)*
Friends	*28 (22.0)*
Social media	*23 (18.1)*
Information evening hospital	*21 (16.5)*
Family	*16 (12.6)*
Magazines	*11 (8.7)*
Pharmacy	*11 (8.7)*
Other	*8 (6.3)*
Digital/media sources	*105 (82.7)*
Professional sources	*85 (66.9)*
Informal sources	*33 (26.0)*

Multiple responses were permitted; therefore, percentages exceed 100%. Percentages are calculated using the total study population as denominator (*n* = 127). Participants reporting more than one information source were counted once for each selected source. No hierarchical assignment of information sources was performed

Exploratory associations between reported information sources and omega-3 supplementation outcomes are presented in Table 5. Participants reporting midwives as a source of information about dietary supplements had higher estimated odds of current omega-3 use than participants not reporting midwives as an information source (OR 6.84, 95% CI 1.56–62.84; *P* = 0.005). However, the confidence interval was wide, indicating substantial uncertainty regarding the magnitude of the association. For the remaining individual and composite information sources, estimates for current omega-3 use were imprecise, with confidence intervals including the null value.

For preconception omega-3 use, participants reporting digital/media sources had higher estimated odds of preconception use than those not reporting these sources (OR 6.59, 95% CI 1.47–61.13; *P* = 0.007), again with a wide confidence interval. Point estimates above 1 were also observed for several individual sources, including internet (OR 2.53, 95% CI 0.95–7.61), magazines (OR 3.69, 95% CI 0.88–18.31), social media (OR 2.44, 95% CI 0.88–6.82), and information evenings (OR 2.41, 95% CI 0.84–7.05); however, these estimates were imprecise and their confidence intervals included the null value. Given the exploratory nature of the analyses, the multiple partly overlapping information-source variables, and the sparse cells in several comparisons, P values were interpreted descriptively rather than according to a dichotomous threshold for statistical significance.

**Table 5:** Exploratory associations between reported information sources and omega-3 supplementation outcomes.

Information source	Current omega-3 use, OR (95% CI)	*P* value	Preconception omega-3 use, OR (95% CI)	*P* value
Midwife	6.84 (1.56–62.84)	0.005	1.32 (0.53–3.22)	0.644
Gynecologist	0.78 (0.31–1.91)	0.703	0.88 (0.39–1.96)	0.865
Internet	1.16 (0.41–3.07)	0.911	2.53 (0.95–7.61)	0.067
Friends	2.24 (0.68–9.69)	0.240	0.87 (0.31–2.27)	0.939
Family	0.96 (0.26–4.45)	1.000	1.15 (0.32–3.82)	1.000
Books	1.96 (0.75–5.60)	0.201	1.49 (0.66–3.37)	0.391
Magazines	0.85 (0.19–5.31)	1.000	3.69 (0.88–18.31)	0.080
Social media	1.20 (0.38–4.54)	0.975	2.44 (0.88–6.82)	0.091
Information evening hospital	1.45 (0.42–6.44)	0.751	2.41 (0.84–7.05)	0.110
Pharmacy	0.85 (0.19–5.31)	1.000	1.09 (0.22–4.57)	1.000
Other sources	0.52 (0.09–3.53)	0.608	1.14 (0.17–6.20)	1.000
Professional sources	0.95 (0.36–2.42)	1.000	0.80 (0.35–1.87)	0.703
Informal sources	1.62 (0.57–5.39)	0.470	0.77 (0.29–1.92)	0.699
Digital/media sources	1.57 (0.48–4.69)	0.525	6.59 (1.47–61.13)	0.007

OR odds ratio, CI confidence interval. ORs are conditional maximum-likelihood estimates with central exact 95% CIs. P values are corresponding two-sided central exact P values. ORs compare participants reporting the respective information source with participants not reporting that source; values > 1 indicate higher odds of the respective omega-3 outcome among participants reporting the source. Each information-source variable was analyzed separately as a binary exposure (reported vs. not reported). Participants could report multiple information sources, and source variables were therefore not mutually exclusive. All source-specific analyses were considered exploratory, and no adjustment for multiple comparisons was applied. Information-source variables refer to sources of information about dietary supplements in general and should not be interpreted as indicating omega-3-specific counseling or recommendations

## Discussion

### Principal findings

The present study investigated omega-3 supplementation during pregnancy, with a particular focus on information sources, educational attainment, and parity. Overall, 75.6% of participants reported current omega-3 supplementation, and 34.6% reported preconception omega-3 use. Internet-based resources represented the most frequently reported source of information, followed by gynecologists and books.

When individual information sources were analyzed separately, participants reporting a midwife as a source of information about dietary supplements had higher estimated odds of current omega-3 supplementation, although the estimate was imprecise. For the remaining individual and composite information sources, estimates for current omega-3 supplementation were imprecise and compatible with a range of possible associations. For preconception omega-3 supplementation, digital/media information sources were associated with higher odds of supplementation before pregnancy, whereas estimates for magazines/flyers, social media, and internet use were above 1, but were imprecisely estimated, as reflected by the wide confidence intervals. No clear association between educational attainment and omega-3 supplementation was observed. Current omega-3 supplementation was descriptively lower across increasing parity categories; however, the number of multiparous participants was small, limiting the precision and interpretation of this pattern. Overall, these exploratory findings indicate co-occurrence between some reported sources of information about dietary supplements and omega-3 supplementation behavior. They do not establish that specific information sources provided omega-3-specific advice, influenced supplementation uptake, or preceded the initiation of supplementation.

### Comparison with existing literature

The prevalence of omega-3 supplementation observed in this study was relatively high, with more than three quarters of participants reporting current supplementation during pregnancy. This finding is broadly consistent with previous studies demonstrating substantial use of nutritional supplements during pregnancy, although reported prevalence varies considerably between populations, healthcare systems, and specific supplements assessed [10–12]. Despite the high overall supplementation rate, only approximately one third of participants reported omega-3 use before pregnancy, suggesting that preconception implementation of nutritional recommendations remains limited.

Internet-based resources represented the most frequently reported information source in our cohort. This finding is consistent with a growing body of evidence demonstrating that pregnant women frequently seek pregnancy-related health information online [20–22]. Recent data from Germany similarly reported that online sources represent one of the most commonly used channels for dietary information during pregnancy [18]. In the present exploratory analysis, reporting the internet as a source of information about dietary supplements was not clearly related to current omega-3 supplementation (OR 1.16, 95% CI 0.41–3.07). The wide confidence interval limits conclusions regarding the presence or magnitude of a possible association. Moreover, the questionnaire did not assess the content of information obtained online or whether it specifically concerned omega-3 supplementation.

Healthcare professionals were also commonly reported as information sources. Previous research has identified healthcare professionals as important providers of nutrition-related information during pregnancy [15]. n the present exploratory analysis, participants reporting midwives among their sources of information about dietary supplements had higher estimated odds of current omega-3 supplementation (OR 6.84, 95% CI 1.56–62.84). However, the wide confidence interval indicates substantial uncertainty regarding the magnitude of this association. No comparable pattern was observed for reporting gynecologists as an information source (OR 0.78, 95% CI 0.31–1.91). Importantly, the questionnaire did not assess whether midwives, gynecologists, or other healthcare professionals specifically discussed or recommended omega-3 supplementation. Recent data from Germany indicate that recommendations regarding omega-3 supplementation vary among gynecologists, potentially contributing to inconsistent patient counselling and implementation [17]. The reasons underlying this association cannot be determined from the present data. Differences in information-seeking behavior, participant characteristics, or unmeasured factors may have contributed to the observed association.

In the exploratory analysis of preconception omega-3 use, participants reporting digital/media sources of information about dietary supplements had higher estimated odds of preconception use (OR 6.59, 95% CI 1.47–61.13), although the wide confidence interval indicates substantial uncertainty regarding the magnitude of this association. Point estimates above 1 were also observed for internet, magazines/flyers, social media, and information evenings, but these estimates were imprecise. This observation is consistent with previous studies suggesting that women planning pregnancy often engage in proactive information-seeking behavior and rely heavily on internet-based resources before their first antenatal consultation [14]. Similarly, Sinikovic et al. [13] reported that books and magazines represent important sources of omega-3-related information among women of reproductive age. However, the present questionnaire assessed sources of information about dietary supplements generally rather than omega-3-specific information, and the timing of exposure to these information sources was not assessed. Therefore, the observed associations cannot establish that exposure to digital/media sources preceded or influenced preconception omega-3 use.

The absence of an association between educational attainment and supplementation behavior contrasts with findings from several previous studies that have reported higher supplement use among women with higher educational attainment [10, 11]. However, the very high educational level observed in the present sample likely reduced variability and limited the ability to detect meaningful differences. These findings suggest that formal educational attainment and health literacy should not be considered equivalent. Previous research has demonstrated that health literacy, information-seeking behavior, and the ability to evaluate health information may be more important determinants of health-related behavior than educational level alone [24–26]. Given the exploratory nature of the source-specific analyses and the multiple comparisons performed, these findings should be interpreted with appropriate caution and require confirmation in larger studies.

### Public health and clinical implications

From a public health perspective, the present findings provide descriptive information on reported information sources and omega-3 supplementation behavior in a selective sample of pregnant women. However, the cross-sectional design does not permit conclusions regarding the effectiveness of specific communication strategies or information channels. As participants could report multiple information sources and important factors such as information quality, trust, frequency of exposure, and temporal relationships were not assessed, the observed associations should be interpreted as exploratory. Future studies using larger samples, multivariable analyses, and preferably longitudinal designs are needed to clarify how different information sources relate to omega-3 supplementation before and during pregnancy [18, 20, 21].

The higher estimated odds of current omega-3 supplementation among participants reporting midwives as a source of information about dietary supplements, and of preconception omega-3 use among those reporting digital/media sources, identify patterns that warrant further investigation. The wide confidence intervals indicate substantial uncertainty regarding the magnitude of these associations. Similarly, the descriptive pattern of lower current supplementation across increasing parity categories requires confirmation in larger samples with greater representation of multiparous women. Future research should also examine factors not assessed in the present study, including health literacy, information quality, and access to understandable evidence-based information.

### Interpretation in a broader context

The findings of this study should be interpreted within the context of an increasingly complex information environment in which pregnant women obtain nutrition-related information from healthcare professionals, digital resources, social networks, and traditional media. In the present exploratory analyses, participants reporting midwives as a source of information about dietary supplements had higher estimated odds of current omega-3 supplementation, whereas participants reporting digital/media sources had higher estimated odds of preconception omega-3 use. Both estimates were accompanied by wide confidence intervals, indicating substantial uncertainty regarding their magnitude.

These findings describe patterns of co-occurrence between reported information sources and supplementation behavior rather than causal relationships. As information sources and supplementation behavior was assessed simultaneously, the temporal sequence of these associations cannot be determined. Furthermore, information quality, source credibility, trust, frequency of exposure, and individual decision-making processes were not assessed. Consequently, the mechanisms underlying the observed associations remain uncertain.

Rather than indicating the superiority of specific information sources, these findings highlight the complexity of information-seeking behavior during pregnancy and identify areas for future research. Larger prospective studies are needed to examine how different information pathways relate to preconception omega-3 use and omega-3 supplementation during pregnancy, including the content, timing, and perceived credibility of the information received.

### Strengths and limitations

Strengths of this study include its focus on omega-3 supplementation during pregnancy, the assessment of reported information sources, and the evaluation of both current supplementation and preconception omega-3 use. To our knowledge, this is among the first studies in Germany to specifically examine associations between reported information sources and omega-3 supplementation behaviour during pregnancy. In addition, the analyses included odds ratios with 95% confidence intervals, with an exact conditional approach used for the exploratory source-specific analyses to account for sparse cells.

Several limitations should be considered. First, participants were recruited through a university hospital and voluntarily completed an online questionnaire, which may have preferentially attracted women who were more health-conscious, digitally engaged, and interested in pregnancy-related health topics. Together with the high educational attainment of the sample, this limits the generalisability of the findings and increases the risk of selection bias.

Second, the relatively small sample size resulted in sparse cells and limited precision in several source-specific analyses, as reflected by the wide confidence intervals. Multiple, partly overlapping information-source variables were examined without adjustment for multiple comparisons. These analyses should therefore be regarded as exploratory, with emphasis placed on the magnitude and precision of the effect estimates rather than on individual P values.

Third, the cross-sectional design precludes conclusions regarding causality or temporal relationships. In addition, all data were self-reported and may therefore be subject to recall and social desirability bias. Because participants could report multiple information sources, the source categories were not mutually exclusive, and information quality, source credibility, trust, frequency of exposure, and temporal sequencing were not assessed. Importantly, the information-source item assessed sources of information about dietary supplements in general rather than sources of omega-3-specific information. It therefore cannot be determined whether any reported source discussed or recommended omega-3 supplementation or whether exposure to a particular source preceded omega-3 use. Consequently, the observed associations should be interpreted as exploratory patterns of co-occurrence rather than evidence of causal relationships or underlying mechanisms.

Finally, the study did not assess dietary omega-3 intake, DHA dosage, duration of supplementation, adherence, or biomarkers of omega-3 status. Residual confounding cannot be excluded, and the findings should therefore be regarded as hypothesis-generating and require confirmation in larger prospective studies.

## Conclusion

In this selective sample of pregnant women, omega-3 supplementation during pregnancy was common, whereas preconception omega-3 use was less frequently reported. In exploratory analyses, participants reporting midwives as a source of information about dietary supplements had higher estimated odds of current omega-3 supplementation, while those reporting digital/media sources had higher estimated odds of preconception omega-3 use. However, both estimates were imprecise, with wide confidence intervals.

These findings represent exploratory patterns of co-occurrence and do not establish that the reported information sources provided omega-3-specific advice, influenced supplementation behaviour, or preceded omega-3 use. Larger, more diverse, and preferably prospective studies are needed to examine these relationships while considering the content and timing of information exposure and potential confounding factors.

## Supplementary Information

Below is the link to the electronic supplementary material.

Supplementary Material 1

Supplementary Material 2

## Data Availability

Data may be made available from the corresponding author upon reasonable request, in accordance with institutional and privacy regulations.
