# Supplementary material for: Omega-3 supplementation during pregnancy in relation to reported information sources: an exploratory cross-sectional study
Source: BMC Pregnancy Childbirth. 2026 Oct 12;26:1151. doi: 10.1186/s12884-026-10082-8 (PMC13656525; doi:10.1186/s12884-026-10082-8)
Supplement: Supplementary file 1 [file 12884_2026_10082_MOESM1_ESM.docx]

# Supplementary Appendix S1

## Demographic characteristics

**1. How old are you?**

- <25 years
- 25–35 years
- 36–45 years
- >45 years

**2. What was your weight at the beginning of your pregnancy?**

_____ kg

**3. What is your current weight?**

_____ kg

**4. What is your height?**

_____ cm

**5. Which week of pregnancy are you currently in?**

_____

**6. What is your highest educational qualification?**

- Lower secondary school certificate (Hauptschule)
- Intermediate secondary school certificate (Realschule)
- Vocational qualification
- University entrance qualification ((Fach-)Abitur)
- Bachelor's degree
- Master's degree or Magister degree
- State examination

**7. How many children have you already given birth to?**

- 0
- 1
- 2
- 3
- More than 3

## Dietary supplements

**8. Are you currently taking dietary supplements?**

- Yes
- No

**9. Have you taken dietary supplements during your pregnancy and subsequently stopped taking them?**

- Yes
- No

**10. Do you know the exact ingredients of the dietary supplement(s) you are taking?**

- Yes
- No

11. **Have you looked into whether dietary supplements could have effects during pregnancy?**

- Yes
- No

**12. Are you taking a combination supplement?**

- Yes
- No

## Omega-3 fatty acids

13. **Are you taking Omega- 3?**

- Yes
- No

**14. Did you take omega-3 supplements before pregnancy?**

- Yes, for more than 3 months
- Yes, for more than 4 weeks
- Yes, starting at the beginning of pregnancy
- Yes, on medical advice since early pregnancy
- No, I do not take omega-3 supplements

**Beliefs and knowledge about omega-3**

**15. Do you believe that your regular diet alone provides sufficient omega-3 for your needs during pregnancy?**

- Yes
- No
- Unsure

**16. What do you consider to be the primary role of omega-3 fatty acids in fetal development?**

- Brain development
- Bone development
- Nervous system development

## Information sources

17. **From which sources did you obtain information about dietary supplements during pregnancy?**
- Midwife
- Gynecologist
- Friends/Acquaintances
- Family
- Internet
- Books
- Magazines/Flyers
- Social media
- Information evening (Hospital)
- Pharmacy
- Other sources
