# Supplementary material for: Omega-3 supplementation during pregnancy in relation to reported information sources: an exploratory cross-sectional study
Source: BMC Pregnancy Childbirth. 2026 Oct 12;26:1151. doi: 10.1186/s12884-026-10082-8 (PMC13656525; doi:10.1186/s12884-026-10082-8)
Supplement: Supplementary file 2 [file 12884_2026_10082_MOESM2_ESM.docx]

# Supplementary Table S1

Coding of questionnaire variables included in the analyses. Question numbers refer to Supplementary Appendix S1.

| Variable analysed | Questionnaire item | Coding |
| --- | --- | --- |
| Age | Q1 | Categorical: <25, 25–35, 36–45, >45 years |
| Pre-pregnancy weight | Q2 | Continuous (kg) |
| Current weight | Q3 | Continuous (kg) |
| Height | Q4 | Continuous (cm) |
| Gestational age | Q5 | Continuous (weeks) |
| Educational level | Q6 | Categorized according to ISCED 2011: Low = ISCED 0–2 (lower secondary school certificate); Medium = ISCED 3–4 (intermediate secondary school certificate, vocational qualification, university entrance qualification); High = ISCED 5–8 (Bachelor's degree, Master's/Magister degree, state examination) |
| Parity | Q7 | 0, 1, 2, 3, >3 live births; categorized as described in the Methods |
| Current dietary supplement use | Q8 | Yes / No |
| Discontinued dietary supplement use during pregnancy | Q9 | Yes / No |
| Knowledge of supplement ingredients | Q10 | Yes / No |
| Looked into possible effects of dietary supplements during pregnancy | Q11 | Yes / No |
| Combination supplement | Q12 | Yes / No |
| Current omega-3 supplementation | Q13 | Yes / No |
| Preconception omega-3 supplementation | Q14 | Derived from Question 14. Participants reporting omega-3 supplementation before pregnancy (>3 months or >4 weeks before pregnancy) were classified as preconception users. All remaining response categories were classified as non-users. |
| Perceived dietary sufficiency of omega-3 | Q15 | Yes / No / Unsure |
| Knowledge of omega-3 function in fetal development | Q16 | Brain development / Bone development / Nervous system development (single-choice) |
| Information source: Midwife | Q17 | Selected / not selected |
| Information source: Obstetrician/Gynecologist | Q17 | Selected / not selected |
| Information source: Friends/Acquaintances | Q17 | Selected / not selected |
| Information source: Family | Q17 | Selected / not selected |
| Information source: Internet | Q17 | Selected / not selected |
| Information source: Books | Q17 | Selected / not selected |
| Information source: Magazines/Flyers | Q17 | Selected / not selected |
| Information source: Social media | Q17 | Selected / not selected |
| Information source: Information evening (Hospital) | Q17 | Selected / not selected |
| Information source: Pharmacy | Q17 | Selected / not selected |
| Information source: Other sources | Q17 | Selected / not selected |

| **Variable analysed** | **Questionnaire item** | **Coding** |
| --- | --- | --- |
| Information source: Professional sources | Q17 | Composite variable. Coded as "selected" if the participant reported at least one of: Midwife, Obstetrician/Gynecologist, or Pharmacy as an information source. |
| Information source: Informal sources | Q17 | Composite variable. Coded as "selected" if the participant reported at least one of: Friends/Acquaintances or Family as an information source. |
| Information source: Digital/media sources | Q17 | Composite variable. Coded as "selected" if the participant reported at least one of: Internet or Social media as an information source. |
